# Shedding light on spawning in jellyfish

**DOI:** 10.7554/eLife.34258

**Published:** 2018-01-22

**Authors:** Laurinda A Jaffe

**Affiliations:** Department of Cell BiologyUniversity of Connecticut Health CenterFarmingtonUnited States

**Keywords:** *Clytia hemisphaerica*, oocyte maturation, photoreception, medusa, reproduction, Other

## Abstract

An opsin receptor has a central role in the production and release of eggs by female jellyfish.

**Related research article** Quiroga Artigas G, Lapébie P, Leclère L, Takeda N, Deguchi R, Jékely G, Momose T, Houliston E. 2018. A gonad-expressed opsin mediates light-induced spawning in the jellyfish *Clytia*. *eLife* **7**:e29555. doi: 10.7554/eLife.29555

For a marine creature about to spawn in the vastness of the ocean, timing is everything. Release gametes before or after everybody else, and chances are the precious cells will drift away without ever encountering their male or female counterparts. In jellyfish, an increase in the amount of sunlight at dawn causes males and females to release sperm and eggs into the water at the same time, therefore improving the chances of fertilization. In female jellyfish, the rise in the amount of sunlight falling on the cells surrounding the oocyte – the future egg – also stimulates the final steps in the process of egg production (Figure 1A; Ikegami et al., 1978; Freeman, 1987). However, the molecular basis of the detection of the light signal has long been a mystery.

**Figure 1.**
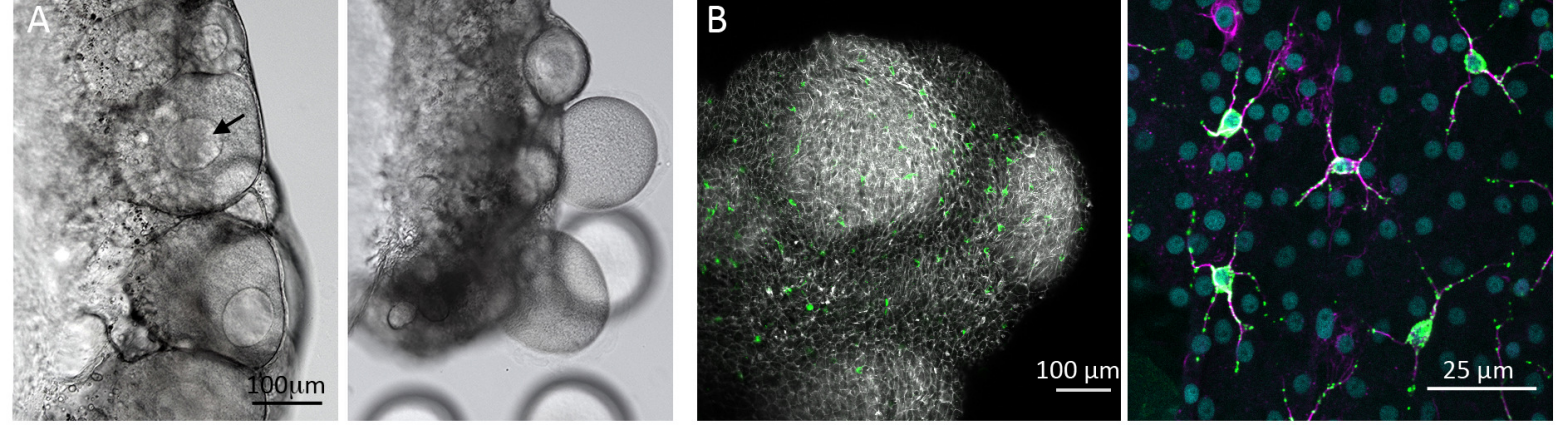
The effect of light on oocytes in the jellyfish *Clytia hemisphaerica*. (**A**) An ovary before (left) and 90 minutes after (right) light stimulation. Before light stimulation the ovary contains resting oocytes (as indicated by the presence of a large oocyte nucleus; arrow), which need to transform into mature eggs for fertilization. Light triggers the breakdown of the nuclear envelope and later the release of mature eggs from the ovary. (**B**) This image of the outer layer of an ovary has a lace-like appearance due to staining of the cell contours in white, with large round oocytes visible behind in grey. In this layer are scattered star-shaped cells that contain both the opsin light receptors and the peptides (labeled in green) that are released from these cells to stimulate the oocytes. In the close-up image on the right, the star-shaped cells are highlighted by staining their characteristic cytoskeleton in pink; the nuclei of the surrounding cells are visible in blue. The left image is about 760 microns across; the right image is about 100 microns across.

IMAGE CREDIT: Evelyn Houliston

Now, in eLife, Evelyn Houliston, Tsuyoshi Momose and colleagues at the Laboratoire de Biologie du Développement de Villefranche-sur-mer – including Gonzalo Quiroga-Artigas as first author and researchers at labs in Japan and Germany – report that they have identified the receptor that performs this role in a species of jellyfish called *Clytia hemisphaerica* (Quiroga Artigas et al., 2018). This protein belongs to the opsin family of receptors, which are responsible for the detection of light throughout the animal world, including in the visual system of vertebrates. Opsins are also involved in the circadian system of many animals (Cermakian and Sassone-Corsi, 2002). Moreover, both the opsin family and the receptors that transmit the signal for oocyte maturation and ovulation in vertebrates are subgroups of a larger family of receptors called G-protein-coupled receptors.

Opsin genes had previously been identified in other jellyfish species, and their expression detected in the gonads of some of these, so there was a good chance that they were involved controlling reproduction. It was also known that the receptor that triggered spawning in response to light was not located in the oocyte itself, but in a layer of cells adjacent to it (Freeman, 1987). While examining gene expression in this location, Quiroga-Artigas et al. found that one opsin (Opsin9) appeared to be very highly expressed in star-shaped cells in the outer layer of the ovary (Figure 1B). Next, they edited *Clytia*’s genome with CRISPR/Cas 9 technology to generate jellyfish lacking Opsin9. These animals failed to release eggs in response to light, thus identifying Opsin9 as the light receptor.

In separate work, Houliston, Ryusaku Deguchi (Miyagi University of Education) and co-workers also discovered that cells expressing Opsin9 produce very short peptides which, when released, act on the oocyte to stimulate maturation and ovulation (Takeda et al., 2017). In jellyfish that lack Opsin9, these peptides are not released, which explains why these animals fail to spawn (Quiroga Artigas et al., 2018). However, the details of the mechanism responsible for the secretion of the peptides, and the details of how these peptides then act on the oocytes, remain to be determined.

Across the animal kingdom, the regulatory pathways that control oocyte maturation all seem to be variations on the jellyfish theme, with G-protein-coupled receptors having central roles. In vertebrates, for example, a G-protein-coupled receptor in the pituitary gland controls the release of luteinizing hormone (Stamatiades and Kaiser, 2017), which then travels through the bloodstream to the ovaries, where it acts on another G-protein-coupled receptor to stimulate the final stage of egg production and ovulation (Jaffe and Egbert, 2017). In birds, light-activated opsins play an active role in coordinating seasonal reproduction, but these receptors are present in the brain, not the ovaries (Halford et al., 2009). Learning more about the ways in which light controls spawning in jellyfish is therefore an important step towards the understanding of the origin and evolution of the processes controlling reproduction.
